# Does a simplified regimen give a better adherence, treatment satisfaction, and quality of life?

**DOI:** 10.7448/IAS.15.6.18091

**Published:** 2012-11-11

**Authors:** N Langebeek, H Sprengers, E Gisolf, C Richter, M Sprangers, P Reiss, P Nieuwkerk

**Affiliations:** 1 Rijnstate Hospital, Department of Internal and Infectious Diseases, Arnhem, Netherlands; 2 University Medical Center, Groningen, Netherlands; 3 Academic Medical Center, Amsterdam, Netherlands

## Abstract

**Background:**

Adherence to treatment is the key to the success of combination antiretroviral therapy (cART) for HIV infection. It is generally assumed that simplification of cART will lead to better adherence, higher treatment satisfaction, and quality of life (QoL), but randomized studies demonstrating such advantages of simplified regimens are scarce.

**Methods:**

Antiretroviral-naïve patients who achieved viral load <50 c/ml in two consecutive samples between 12–24 weeks after commencing induction therapy with bid lopinavir/ritonavir (LPV/r) and fixed-dose combination AZT/3TC (Combivir^®^) were randomly assigned to either continue LPV/r/Combivir^®^ or switch to simplified therapy with bid fixed-dose AZT/3TC/abacavir (Trizivir^®^). Both arms yielded similar antiviral efficacy after 48 weeks as reported previously [1]. Patients completed standardized questionnaires on adherence (Simplified Medication Adherence Questionnaire (SMAQ)), treatment satisfaction (HIVTSQ) and QoL (MOS-HIV) at randomization and at weeks 48, 72 and 96. Adherence data were analyzed using generalized estimating equations, and satisfaction and QoL using mixed linear models.

**Results:**

Patients in the Trizivir^®^-group (n=30) tended to skip fewer doses both during the preceding 7 days (p=0.055) and during the preceding weekend (p=0.09) than patients in the LPV/r-group (n=20). Moreover, patients in the Trizivir^®^-group found their regimen significantly more convenient (p=0.022) than patients in the LPV/r-group. However, patients in the LPV/r-group reported a better QoL than patients in the Trizivir^®^-group in the domains measuring cognitive functioning (p=0.003), energy/fatigue (p=0.046) and social functioning (p=0.074).

**Conclusions:**

In this randomized trial simplification of therapy to fixed-dose Trizivir^®^ was perceived to be more convenient, and tended to result in improved adherence, but at the expense of a lower level of QoL. Our findings suggest that the choice for simplified regimens should be individualized and may involve a trade-off between convenience and QoL.
